# Organometallic gold(I) and gold(III) complexes for lung cancer treatment

**DOI:** 10.3389/fphar.2022.979951

**Published:** 2022-09-13

**Authors:** Juzheng Zhang, Yanping Li, Ronghao Fang, Wei Wei, Yong Wang, Jiamin Jin, Feng Yang, Jian Chen

**Affiliations:** ^1^ Guangxi Key Laboratory of Tumor Immunology and Microenvironmental Regulation, Guilin Medical University, Guilin, China; ^2^ School of Public Health, Guilin Medical University, Guilin, China; ^3^ State Key Laboratory for Chemistry and Molecular Engineering of Medicinal Resources, Collaborative Innovation Center for Guangxi Ethnic Medicine, School of Chemistry and Pharmaceutical Sciences, Guangxi Normal University, Guilin, China

**Keywords:** Gold(I) complexes, gold(III) complexes, anticancer, lung cancer, A549

## Abstract

Metal compounds, especially gold complexes, have recently gained increasing attention as possible lung cancer therapeutics. Some gold complexes display not only excellent activity in cisplatin-sensitive lung cancer but also in cisplatin-resistant lung cancer, revealing promising prospects in the development of novel treatments for lung cancer. This review summarizes examples of anticancer gold(I) and gold (III) complexes for lung cancer treatment, including mechanisms of action and approaches adopted to improve their efficiency. Several excellent examples of gold complexes against lung cancer are highlighted.

## GRAPHICAL ABSTRACT

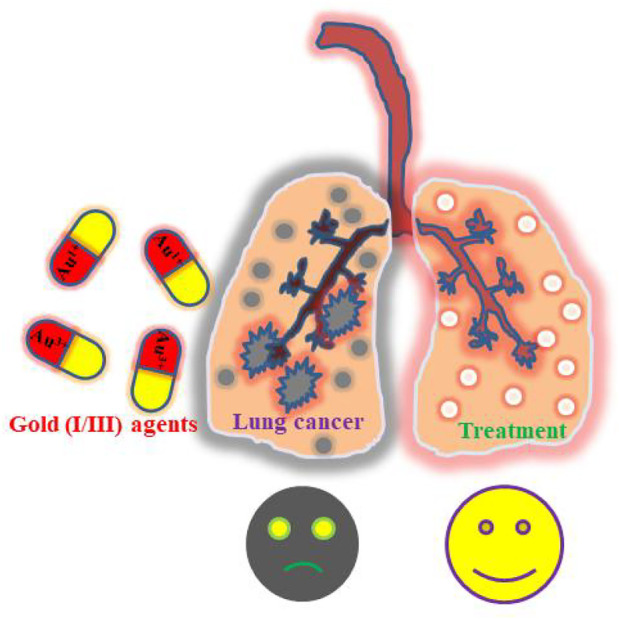

## Introduction

Cancer, especially lung cancer, ranks as a leading cause of death worldwide (Boshoff et al., 2018; Cheng et al., 2020; Boshoff et al., 2021; Hanna and Miller, 2021; Heist et al., 2021; Tan et al., 2021). The 2020 global cancer statistics covering 185 countries show that lung cancer remained the leading cause of cancer mortality, with an estimated 1.8 million deaths (18% of all cancer) (Bray et al., 2021). Thus, efforts to develop novel anti-lung cancer drugs and strategies are critical for the global control of this disease.

**FIGURE 1:**
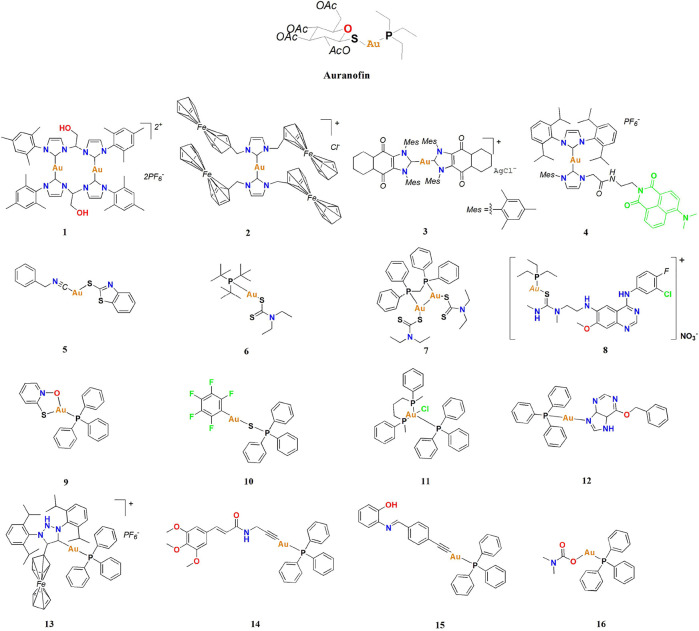
Anti-lung cancer gold(I) complexes.

Metal compounds, especially gold complexes, have recently gained increasing attention in the design of lung cancer therapeutics (Grapperhaus et al., 2020; Akbarsha et al., 2021; Zhang et al., 2021a; Chao et al., 2021; Daher et al., 2021). Gold complexes are a class of compounds formed by ligand coordination with gold(I) or gold (III) ions, which contain nitrogen, phosphorus, sulfur, carbon, and other atoms. The geometric configuration of gold complexes is changeable, mostly in compositions of two, three, and four ligands (Che et al., 2017; Bourissou et al., 2020; Gornitzka et al., 2020; Ott et al., 2020; Zou et al., 2020, 2021, 2022). Gold complexes also have unique electronic structures and changeable redox states responsible for their excellent catalytic performance, rich optical properties, and notable biological activities. Therefore, they have broad application prospects as catalysts, optical materials, protein inhibitors, and anticancer drugs (Dou et al., 2010; Pizarro et al., 2018; Arambula et al., 2020; Liu et al., 2020; Dos et al., 2021; Rodriguez-Yoldi et al., 2021). With the discovery of auranofin’s anticancer activity (Figure 1) (Abdalbari and Telleria, 2021), a new chapter exploring gold complexes as anticancer drugs was opened. Researchers have successively modified and improved the ligands bound to gold ions and synthesized many gold complexes with anticancer activity, including gold(I) and gold (III) (Liu et al., 2022).

In recent years, gold complexes have gained increasing attention in the search for lung cancer treatments. Many anti-lung cancer gold(I) and gold (III) complexes have been designed and synthesized for lung cancer treatments (Fregona et al., 2018; Bostancıoğlu et al., 2019; Alonso et al., 2020; Gianneschi et al., 2021; Pawlak et al., 2021). These complexes contain multidentate N-donor, cyclometalating, dithiocarbamate, triazole–peptide, N-heterocyclic carbine (NHC), thiourea, alkynyl, thiolate, phosphine, or other ligands (Eichler et al., 2014; Kühn et al., 2017; Fereidoonnezhad et al., 2020; Marchetti et al., 2022). This review summarizes the development of gold complexes as anti-lung cancer agents, including their mechanisms against lung cancer and the approaches adopted to improve their anti-lung cancer efficiency. In particular, several examples which exhibit excellent anti-lung cancer effects *in vivo* are highlighted.

## Anti-lung cancer gold(I) complexes

**FIGURE 2:**
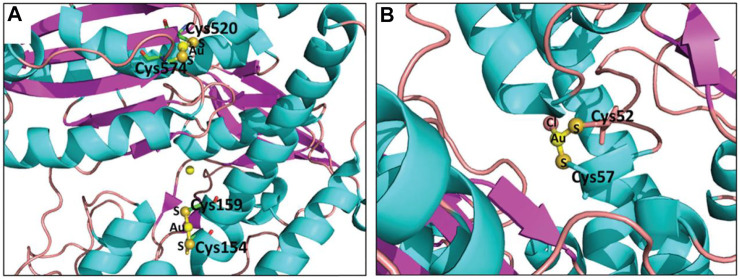
Crystal structure of gold(I)-protein conjugates: **(A)** Au(I)-TGR conjugates (PDB code: 3H4K, gold source: auranofin). **(B)** Au(I)-TR conjugates (PDB code: 2YAU, gold source: auranofin). Reproduced with permission (Bellelli et al., 2009; Colotti et al., 2012).

Auranofin, a well-known gold(I)–phosphine–thiolate complex, was developed to treat rheumatoid arthritis in a clinical setting (Furst, 1983; Avery et al., 1984; Shea et al., 2000; Kast, 2010; Choi et al., 2021). Recently, auranofin was evaluated as an agent to inhibit the growth of different cancers (Fang et al., 2018; Kawada et al., 2019; Wang et al., 2020; Abdalbari and Telleria, 2021; Kawada et al., 2021), including lung cancer (IC_50_ < 2 μM, for A549). Research showed that auranofin is an effective selective inhibitor of thioredoxin reductase (TrxR) by forming a linear Scys-Au^I^-Scys coordination bond with TrxR (Figure 2) (Bellelli et al., 2009; Colotti et al., 2012). It can induce the generation of reactive oxygen species (ROS) and activate p38 mitotic activated protein kinase (p38 MAPK) (Park and Kim, 2005). The latest research found that auranofin may exert its anticancer effect by inhibiting proteasome-associated deubiquitinases (DUBs). Liang and co-workers found that inhibiting TrxR would lead to 36% Cys oxidation of 606 Cys-containing peptides. These studies prove that auranofin can effectively induce oxidation of Cys peptides and inhibit TrxR activity (Jones et al., 2013; Liu et al., 2014). The success of auranofin as a cancer treatment has aroused the interest of pharmaceutical chemists in other gold(I) complexes (Liu et al., 2022).

### Anti-lung cancer gold(I)-NHC complexes

**FIGURE 3:**
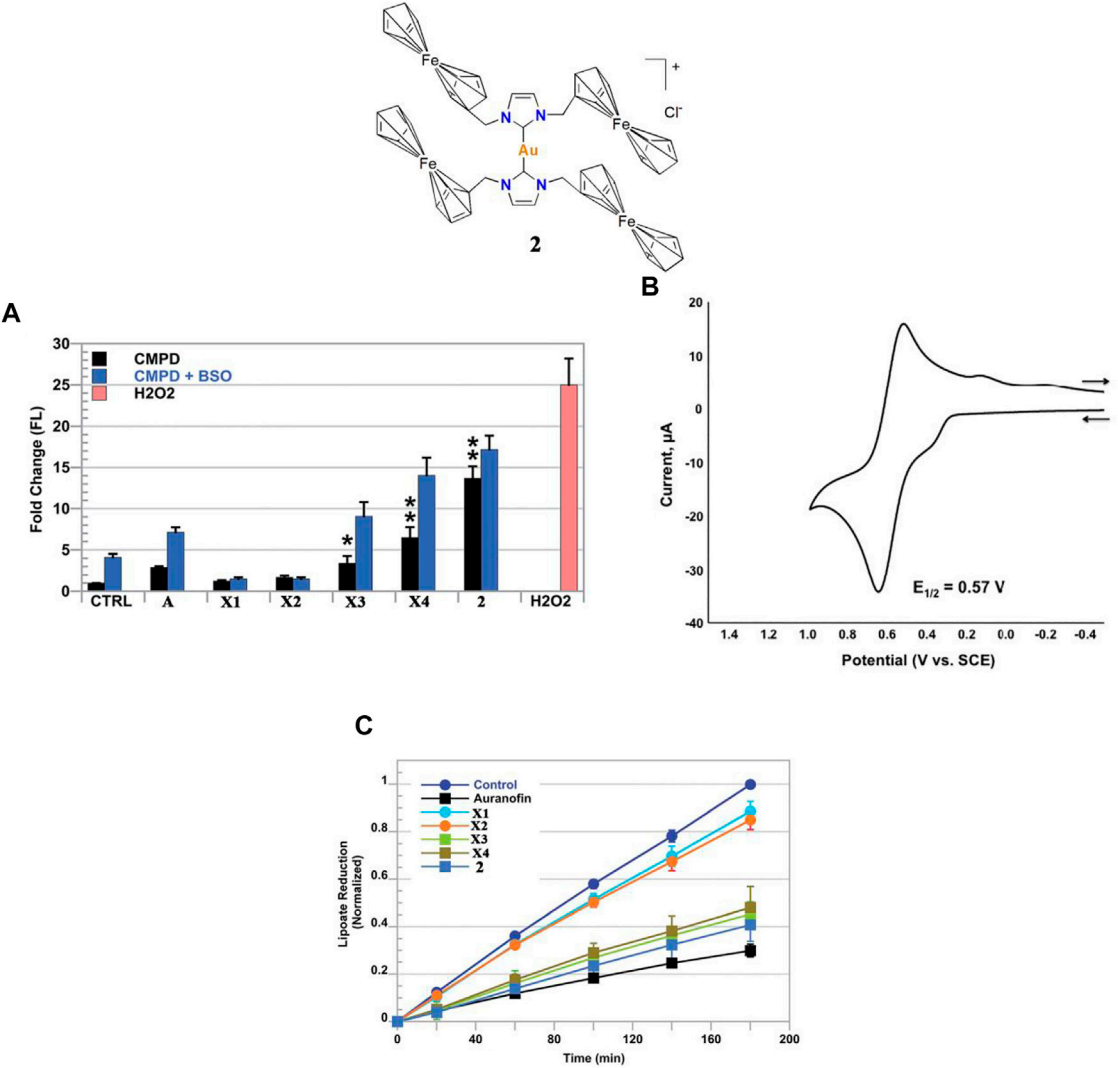
**(A)** ROS detected by fluorescent signal increases DCF via flow cytometric analysis in live A549 cells treated with complex **2**. H_2_O_2_ was used as a positive control. **(B)** CV (100 mV s^−1^ scan rate) and DPV (50 mV pulse amplitude) of complex **2**. **(C)** Time-dependent inhibition of thioredoxin reductase (TrxR) via the reduction of lipoate (*p < 0.05; **p < 0.01). Reproduced with permission (Arumugam et al., 2016).

Kühn and co-workers investigated gold(I)-NHC complex **1**. After a 48 h treatment, complex **one** showed high IC_50_-values in an A549 cell line, which may be due to the low solubility of complex **one** in aqueous medium (Kühn et al., 2017). Arumugam and co-workers investigated ferrocenylated N-heterocyclic carbene-supported gold(I) complex **2**, the cytotoxicity of **2** was found to be tenfold greater than auranofin in the A549 cell line (Arumugam et al., 2016). In addition, Arumugam and co-workers found that ferrocene significantly enhanced the cytotoxicity of gold(I)-NHC complexes in the A549 cell line. Although the *in vivo* anti-lung cancer activity of complex **2** was not described in this literature, Arumugam and co-workers have confirmed that complex **2** kills A549 cells by a dual-mode of action, inducing ROS generation (Figures 3A,B) and TrxR inhibition (Figure 3C).

**FIGURE 4:**
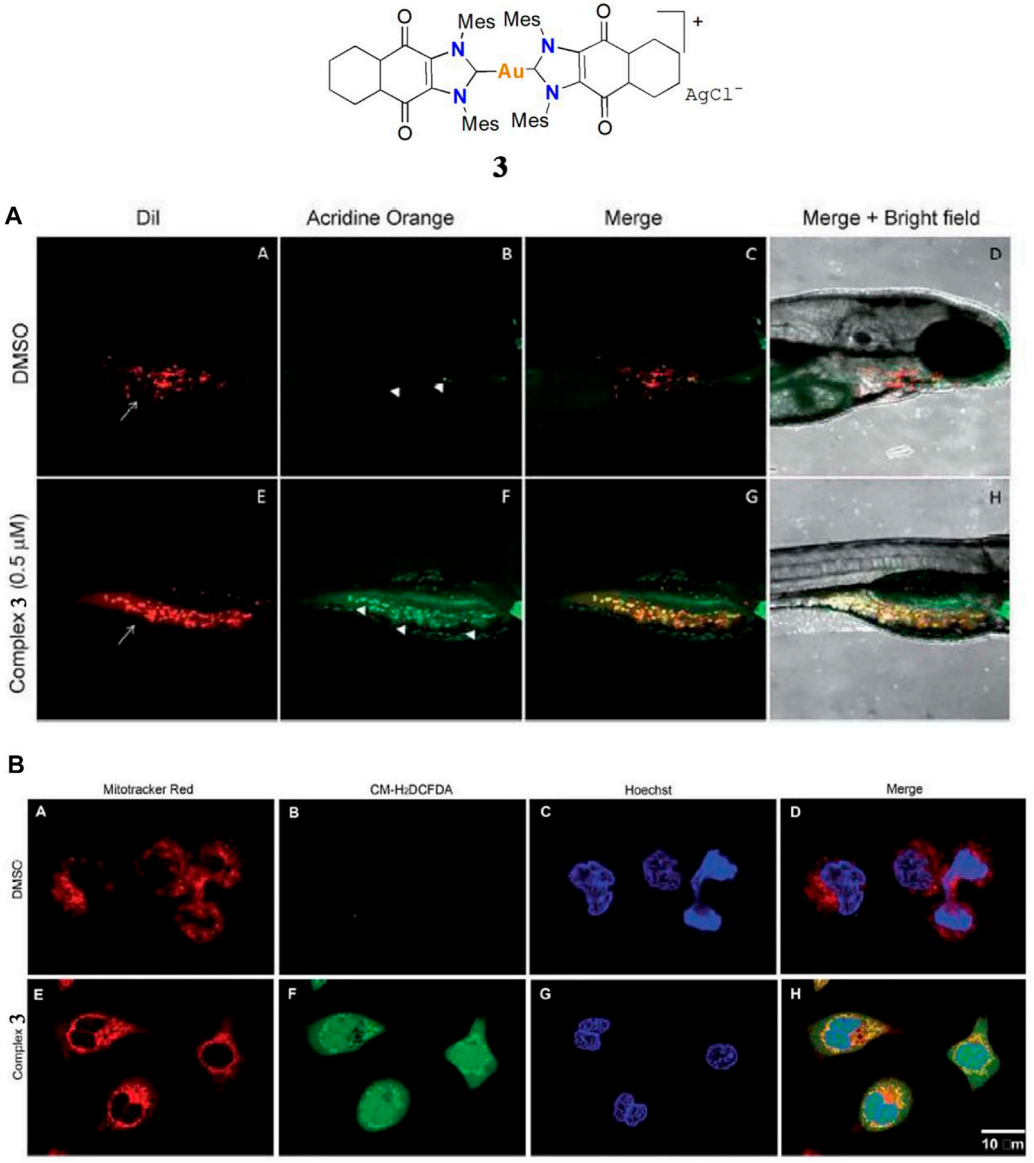
**(A)** Complex **3** against A549 cells in a Zebrafish tumor xenograft model. **(B)** Confocal microscopy images of mitochondria ROS generation after treatment with 1.25 μM of complex **3** in the A549 cell line. Reproduced with permission (Arambula et al., 2017).

In 2017 Arambula and co-workers investigated a gold(I)-NHC complex **3** with excellent cytotoxicity in the A549 cell line; the IC_50_ value was 0.07 μM (Arambula et al., 2017). Zebrafish-A549 embryos as a tumor xenograft model were treated with complex **3** at a concentration of 0.5 μM for 72 h. Tumor inhibition was determined by acridine orange staining. Apoptotic cells show a significant increase in yellow or orange color. At a dose of 0.5 μM, complex **3** displayed low toxicity and excellent tumor inhibition activity (Figure 4A), indicating it is worthwhile to consider it for further study as an anti-lung cancer agent. Like complex **2**, the anticancer mechanism of complex **3** is mainly via ROS generation by redox cycling (Figure 4B) and TrxR inhibition.

**FIGURE 5:**
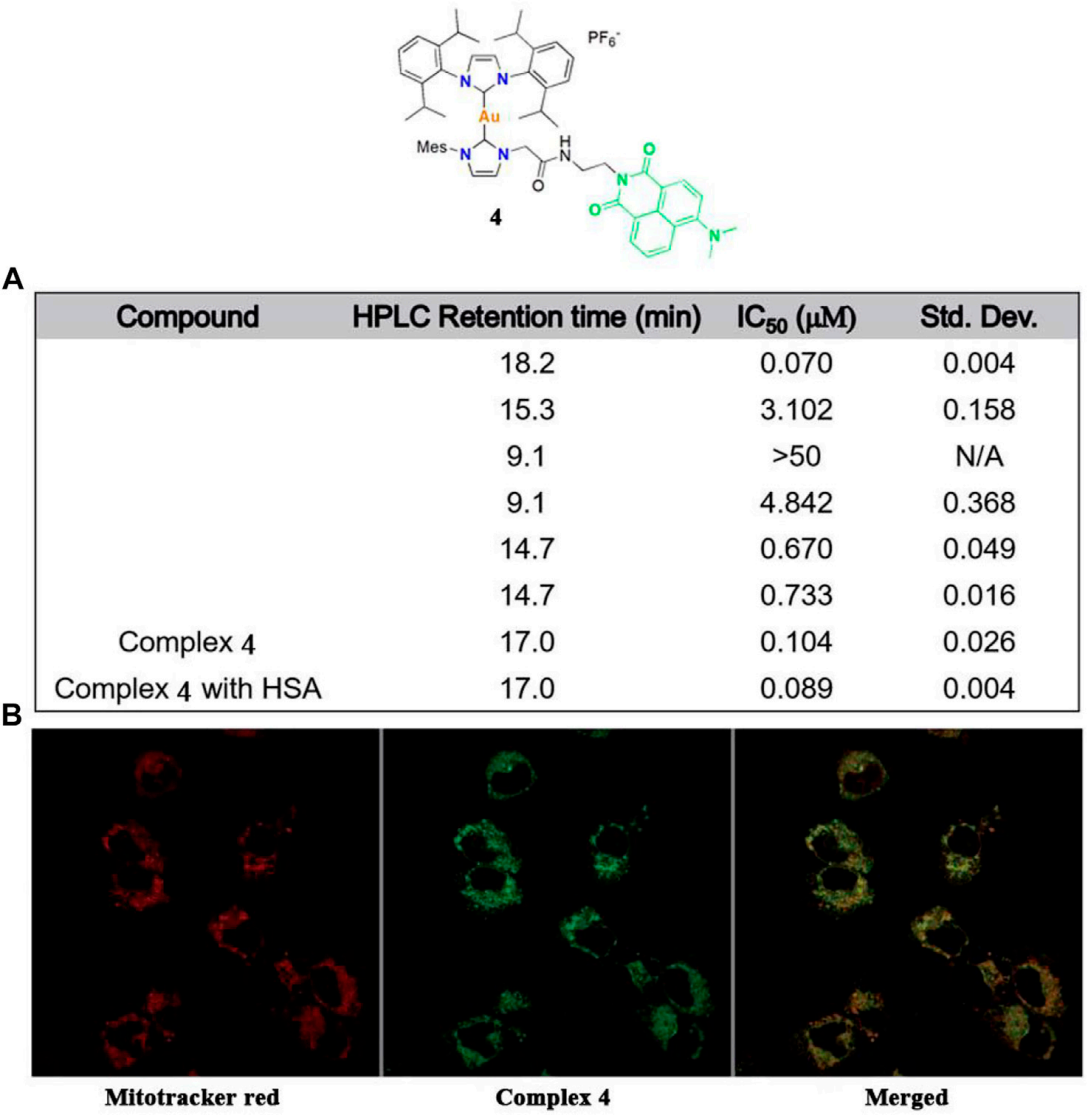
**(A)** Cytotoxicity values (IC_50_, μM) of complex **4** against A549 cells by MTT assays. **(B)** Confocal microscopy images obtained after treatment with complex **4** in the A549 cell line. Reproduced with permission (Arambula et al., 2021).

Arambula and co-workers modified a gold(I)-NHC complex **4** in 2021 (Figure 5A), which could also be effectively tracked by confocal microscopy (Figure 5B) (Arambula et al., 2021). Intracellular tracking experiments showed that complex **4** accumulated in the mitochondria, causing oxidative stress and promoting ROS production. In addition, human serum albumin can significantly enhance the solubility of complex **4** in water. Studies focused on the rationally designed gold(I)-NHC complex via non-covalent binding with serum proteins for tumor targeting. The resulting complex **4** retained strong cytotoxicity and a therapeutic effect.

### Anti-lung cancer gold(I)-sulfur complexes

Auranofin, gold(I)-sulfur complexes have been studied as anticancer complexes for more than 20 years (Avarvari et al., 2008; Silva et al., 2016; de Almeida et al., 2017; Du et al., 2017; Gimeno et al., 2018; Liu et al., 2020). Recently, these gold(I)-sulfur complexes have been investigated as lung cancer treatments. Fereidoonnezhad and co-workers reported a novel gold(I)-sulfur complex **5** that appeared to have moderate anti-proliferative effects in the A549 cell line (Fereidoonnezhad et al., 2020). The mechanism of action is slightly different from that previously reported in that molecular docking analysis confirmed that complex **5** affected A549 cells by inhibiting TrxR and intercalation of DNA. Dhuna and co-workers reported a gold(I)-sulfur complex **6** (Dhuna et al., 2015); however, the mechanism of action was not reported in this case.

Complex **7**, reported by Korashy and co-workers, exhibited much better anti-lung cancer potency than cisplatin (Korashy et al., 2018). They constructed a biological network of miRNA and gene targets for up and downregulated miRNAs to study the effect of gold complex-altered miRNA expression profiles in A549 cells. Their studies show that complex **7** can disrupt the basic cellular mechanisms in A549 cell lines by affecting the micro-RNA network. The importance of developing new gold(I) complexes and their potential as new anti-lung cancer therapeutic agents were clarified.

Gefitinib is an epidermal growth factor receptor tyrosine kinase (EGFR-TK) inhibitor for non-small cell lung cancer treatment (Kirkpatrick et al., 2003; Yoshida et al., 2009; Inoue et al., 2020; Novello et al., 2020; Gibbons et al., 2021; Wu et al., 2021). Bierbach and co-workers utilized thiourea-modified gefitinib to derive linear gold(I)-sulfur complex **8**. The cytotoxicity of complex **8** was also studied in lung cancer cells NCI-H460 (IC_50_ = 1.9 μM) (Bierbach et al., 2015). Compound **8** inhibited EGFR kinase-mediated phosphorylation, and its submicromolar IC_50_ value was similar to gefitinib observed under the same analytical conditions. Extension of the side chain on carbon 6 of the quinazoline ring to generate **8** led to a pronounced increase in potency. Compound **8** alone showed significantly better activity than gefitinib in a TKI-resistant cancer cell line. This observation suggests that the newly introduced thiourea-containing side chain may enhance the binding affinity of the classical TKI structure with the enzyme’s active site. This new type of metal hybrid agent designed by combining a biomolecular targeting inhibitor and metal ions provides new inspiration for the development of novel anticancer drugs.

**FIGURE 6:**
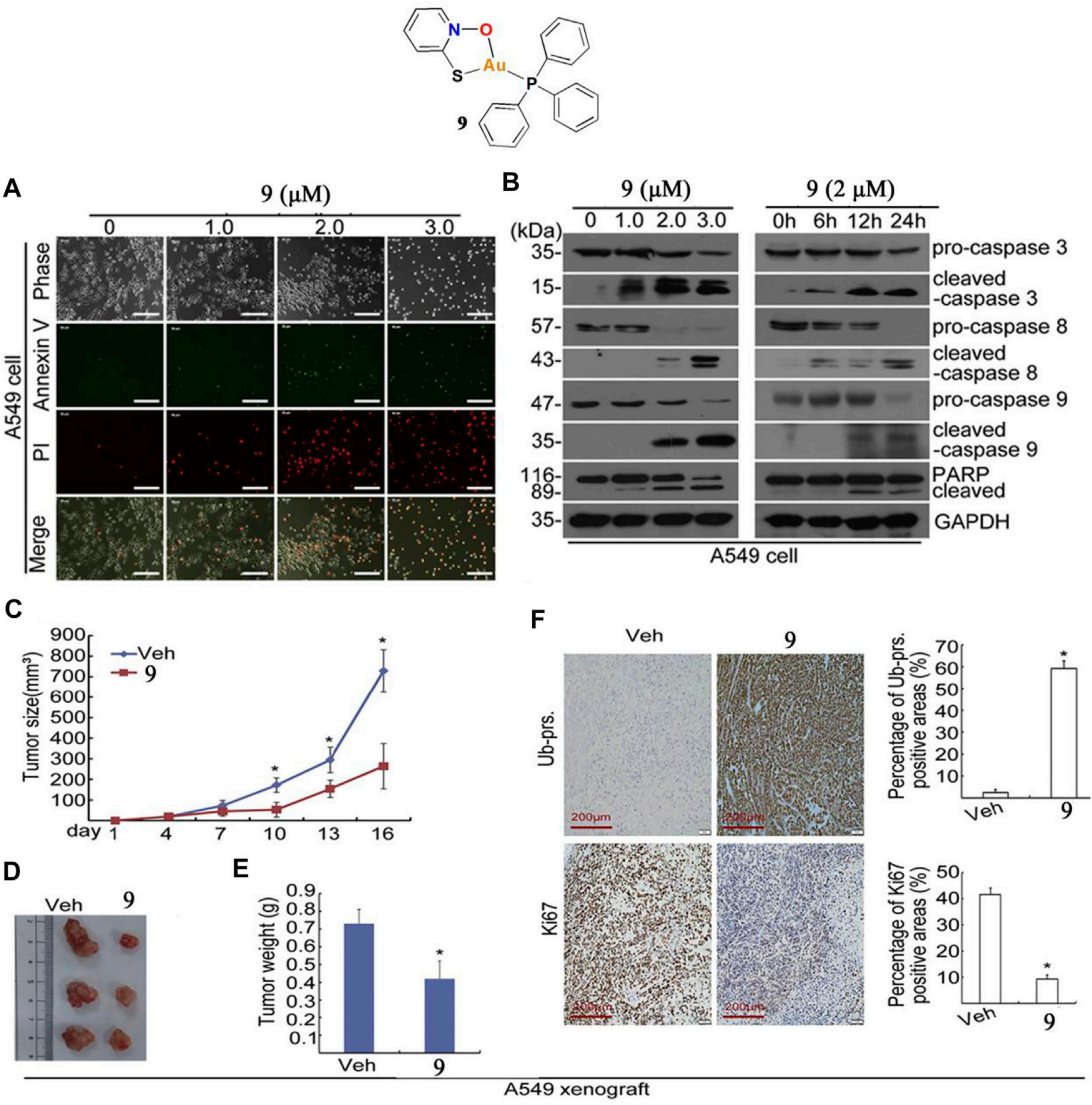
**(A)** Apoptotic cells were detected by Annexin V-FITC/PI staining after treatment with complex **9**. **(B)** Western blot analysis of caspase-3, 8, and nine in A549 cells after treatment with complex **9**. **(C–F)** BALB/c nude mice bearing A549 tumors after treatment with complex **9** (7 mg/kg/day) for 16 days. Reproduced with permission (Liu et al., 2019).

Liu and co-workers developed a gold(I)-sulfur-phosphine complex **9** as a deubiquitinase inhibitor to prevent lung cancer A549 tumor growth (Liu et al., 2019). The cytotoxic effects of complex **nine** in lung cancer cells A549 were determined by MTT assay at different concentrations for 24, 48, or 72 h. The results indicated that complex **nine** significantly inhibited A549 cell viability after the 72 h treatment. In addition, Liu and co-workers also determined the complex **9**-induced A549 cell death mechanism. There was a dose-dependent increase in AnnexinV/PI-positive cells after treatment with complex **nine** for 24 h. Western blot analysis showed that complex **nine** significantly increased the cleaved forms of caspase-3, 8, and nine to induce A549 cell apoptosis (Figures 6A,B). Like others gold complexes (**1**–**5**), complex **nine** also increased ROS production in A549 cells. However, the production of ROS induced by complex **nine** does not play a major role in apoptosis of A549 cells. Liu and co-workers used BALB/c nude mice to create xenograft models and assess the anti-lung cancer tumor action of complex **nine** *in vivo*. Treatment with complex **nine** via intraperitoneal injection (7 mg/kg/day) for 16 days in the A549 xenograft model showed significant inhibition of tumor growth, which led to a reduction in tumor weight of ∼42.5% (Figures 6C–F). Furthermore, the mice did not experience significant body weight loss after injection of complex **9**.

Alonso and co-workers reported gold(I)-sulfur-phosphine complex **10** as an excellent cytotoxicity gold(I) complex against lung cancer cell line A549 with an IC_50_ value of 0.03 μM (Alonso et al., 2020). The high cytotoxicity of complex **10** was not due to an increase in ROS content in cells but through inhibition of topoisomerase I. Unfortunately, the *in vivo* anti-lung cancer activity of complex **10** was not described in the literature.

### Anti-lung cancer gold(I)-phosphine complexes

Metal-phosphine complexes have been utilized as cancer therapeutics for decades (Marzano et al., 2010; Bhargava et al., 2018; Awuah et al., 2019; Sitole et al., 2020; Batista et al., 2021; Tavares et al., 2021; Liu et al., 2022). In 2003, Marchetti and co-workers described a mixed phosphine gold(I) complex **11** as a potent anticancer agent (Marchetti et al., 2003), with positive anti-proliferative effects in the A549 cell line. The possibility exists for tuning the antitumor activity of gold-diphosphine cationic compounds between these two extremes: 1) more lipophilic complexes which are more cytotoxic and have increased side effects on mitochondria; 2) more hydrophilic compounds which are more selective, less cytotoxic, and have lower side effects. Unfortunately, the *in vitro* anti-lung cancer mechanism of complex **11** was not described in the literature. In 2014, Trávníček and co-workers modified a gold(I)-triphenylphosphine complex **12** with a hypoxanthine derivative as an anticancer agent (Trávníček et al., 2014). The IC_50_ values for the tested complex **12** showed moderate activity in the micromolar range.

**FIGURE 7:**
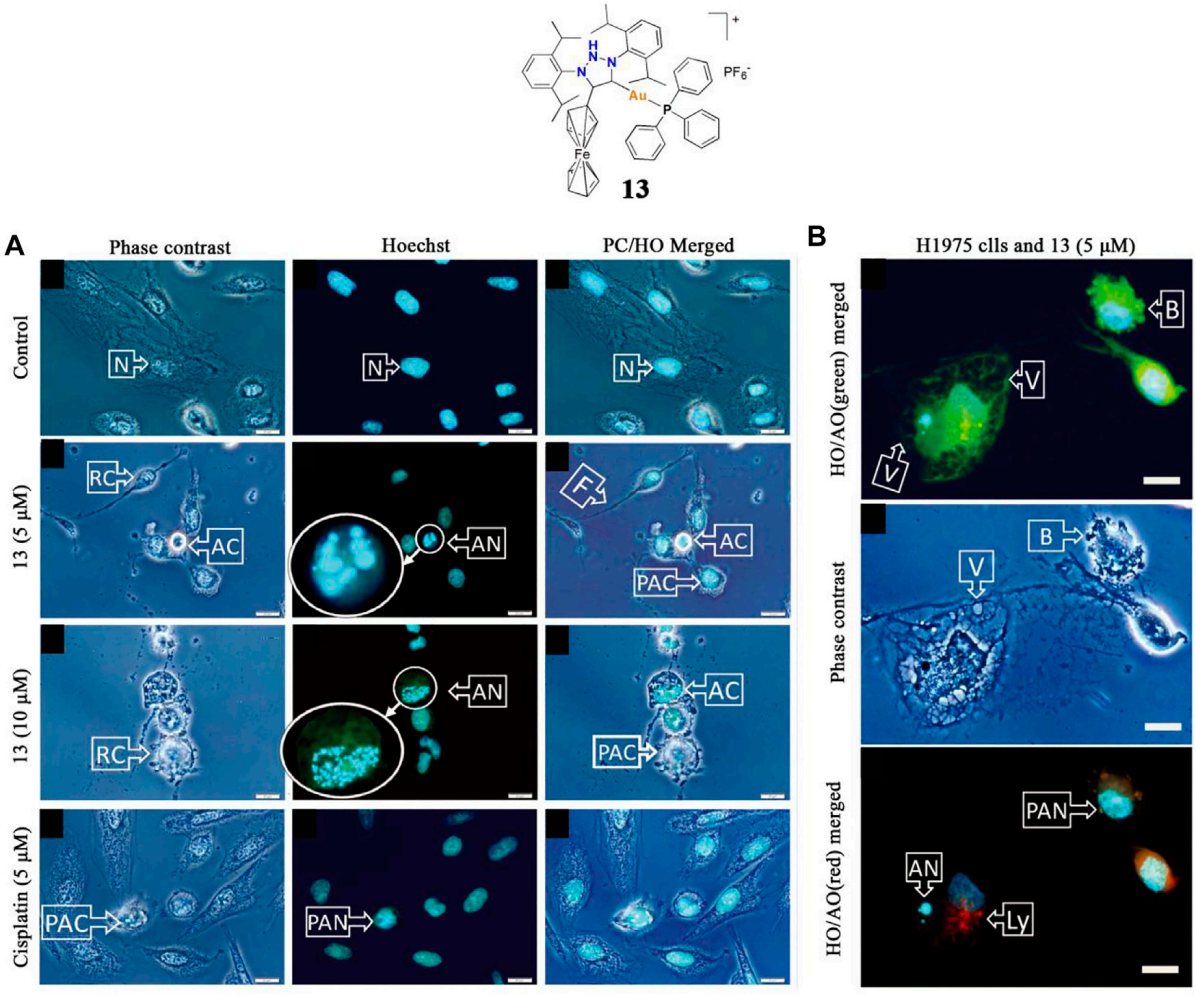
**(A)** Lung cancer cells H1975 undergo apoptosis after being treated with complex **13** at 5/10 μM concentrations for 18 h **(B)** Microscopic evaluation of H1975 cell morphology after treatment with complex **13** at a 5 μM concentration for 18 h. Reproduced with permission (Bezuidenhout et al., 2018).

In 2018, Bezuidenhout and co-workers identified a gold(I)-phosphine-ferrocenyl substituted 1,2,3-triazol-5-ylidene complex **13** as a potential anticancer agent (Bezuidenhout et al., 2018). The anticancer activity of complex **13** was assessed against two lung cancer cell lines, A549 and H1975, and the IC_50_ values were 0.89 and 0.23 μM, respectively. This complex **13** exhibited much higher anti-lung cancer activity than cisplatin. Fluorescence microscopy confirmed that gold(I) complex **13** induced H1975 lung cancer cell death by apoptosis but was not necrotic (Figures 7A,B), and preliminary judgment indicated that ROS played a key role in mediating cell death.

**FIGURE 8:**
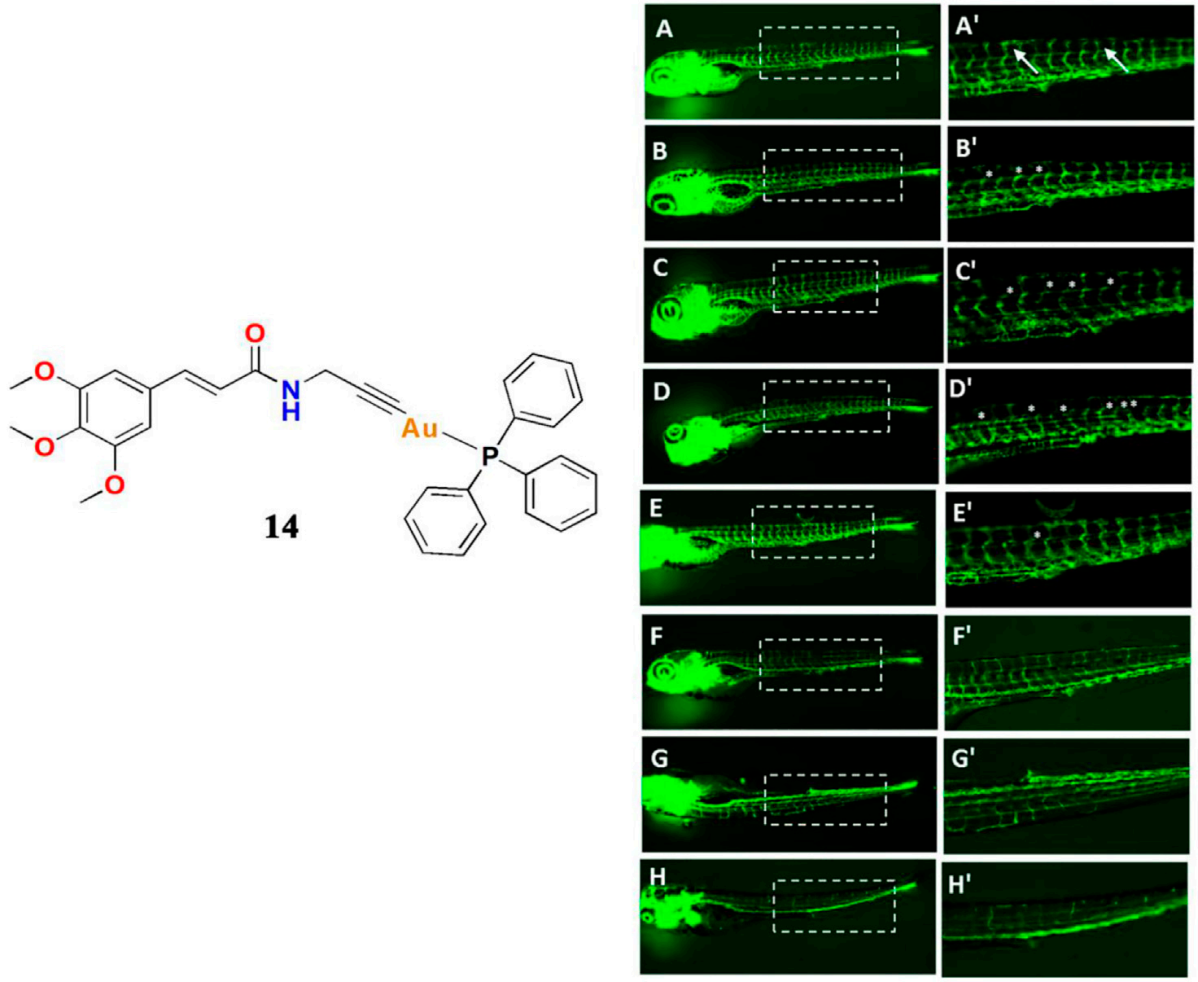
Angiogenesis inhibition in transgenic Tg (fli1a:EGFP) zebrafish embryos after treatment with different concentrations of complex **14**, with cisplatin and axitinib as positive controls, at 24 hpf. **(A, A′)** control **(B−D, B′−D′)** complex **14** at 0.1/0.25/0.5 μM **(E,E′)** cisplatin (1 μM) **(F−H, F′−H′)** axitinib at 0.1/0.2/0.5 μM concentrations. Reproduced with permission (Bhargava et al., 2019).

In 2019, Bhargava and co-workers reported a gold(I)-phosphine Ph3 containing cinnamide and alkynyl complex **14** (Bhargava et al., 2019). The results clearly indicate that the gold(I) alkynyl fragment enhances the anticancer activity, and the cytotoxicity of the compounds was also influenced by the number of methoxy groups present in the alkyne moiety. They also confirmed that complex **14** has significant antiangiogenic effects. The anticancer mechanism involved is similar to that of most gold(I) complexes in that complex **14** significantly inhibits TrxR and increases ROS accumulation. Sordillo and co-workers discovered that TrxR regulates angiogenesis by increasing endothelial cell-derived vascular endothelial growth factor leading Bhargava and co-workers to consider that complex **14** could be contributing to the inhibition of angiogenesis. Thus, they investigated the angiogenesis-inhibiting properties of complex **14** using a transgenic Tg (fli1a:EGFP) zebrafish model (Figure 8). Their results indicated that complex **14** had significant antiangiogenic effects in zebrafish embryos and that gold(I) complexes may potentially become promising cancer therapeutics.

Hussien and co-workers investigated gold(I)-phosphine Ph3 containing Schiff bases and alkynyl complex **15** in 2021 (Hussien et al., 2021) as an anti-proliferative in lung cancer cell line HOP-62. Like most gold(I) complex investigations, Hussien and co-workers also focused on inhibiting the redox enzyme TrxR by complex **15**. They used molecular docking to study the gold(I) complexes in the two active sites of the human TrxR enzyme, using auranofin as a benchmark for comparison. They found that introducing the Schiff base phenolic moieties can alter the binding sites of the gold(I) complexes with TrxR, unlike auranofin. Complex **15** seems to be more cytotoxicity against lung cancer when compared to the other complexes, indicating a possible preference for the ortho position for the hydroxy group.

**FIGURE 9:**
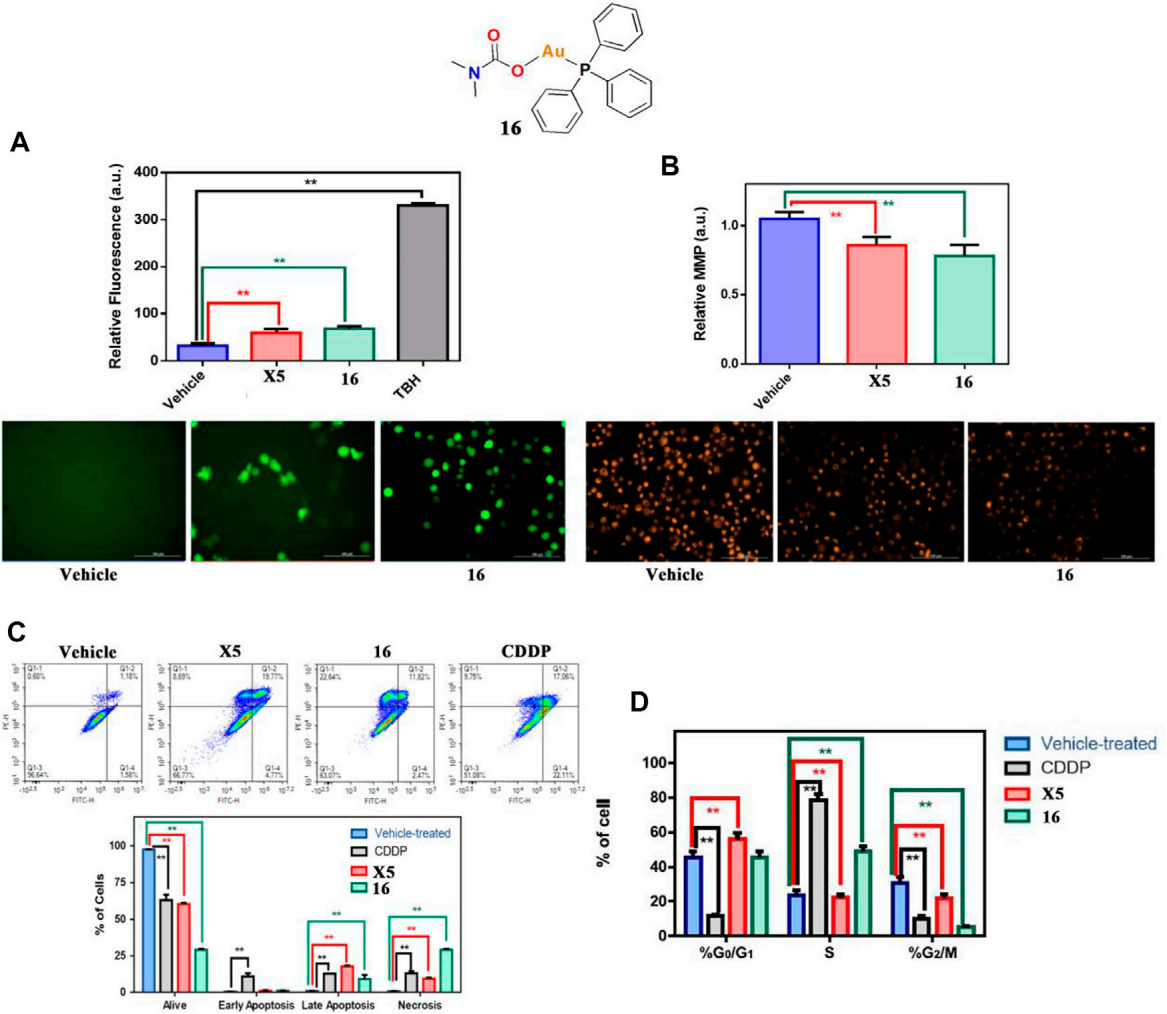
**(A)** ROS production by H_2_DCFDA in A549 cells after treatment with complex **16** at IC_50_ values for 4 h. **(B)** Mitochondrial membrane potential by TMRM in A549 cells after treatment with complex **16** at IC_50_ values for 1 h. **(C)** Flow cytometer analysis of A549 cells treated with complex **16** at IC_50_ values for 24 h. **(D)** Flow cytometer analysis of A549 cell cycle treated with complex **16** at the IC_50_ value for 24 h. Reproduced with permission (Marchetti et al., 2022).

The latest studies indicate that gold(I)-phosphine complexes play an anticancer role other than inhibition of TrxR. In 2022, Marchetti and co-workers described a gold(I)-phosphine Ph3 containing carbamate complex **16** that exhibited positive *in vitro* cytotoxicity for A549 lung cancer cells (Marchetti et al., 2022). Interestingly, Marchetti and co-workers confirmed that complex **16** could significantly increase intracellular ROS accumulation by mitochondrial membrane depolarization (Figures 9A,B) and that it could also induce necroptosis and block cell cycle arrest in S phase (Figures 9C,D).

## Anti-lung cancer gold (III) complexes

**FIGURE 10:**
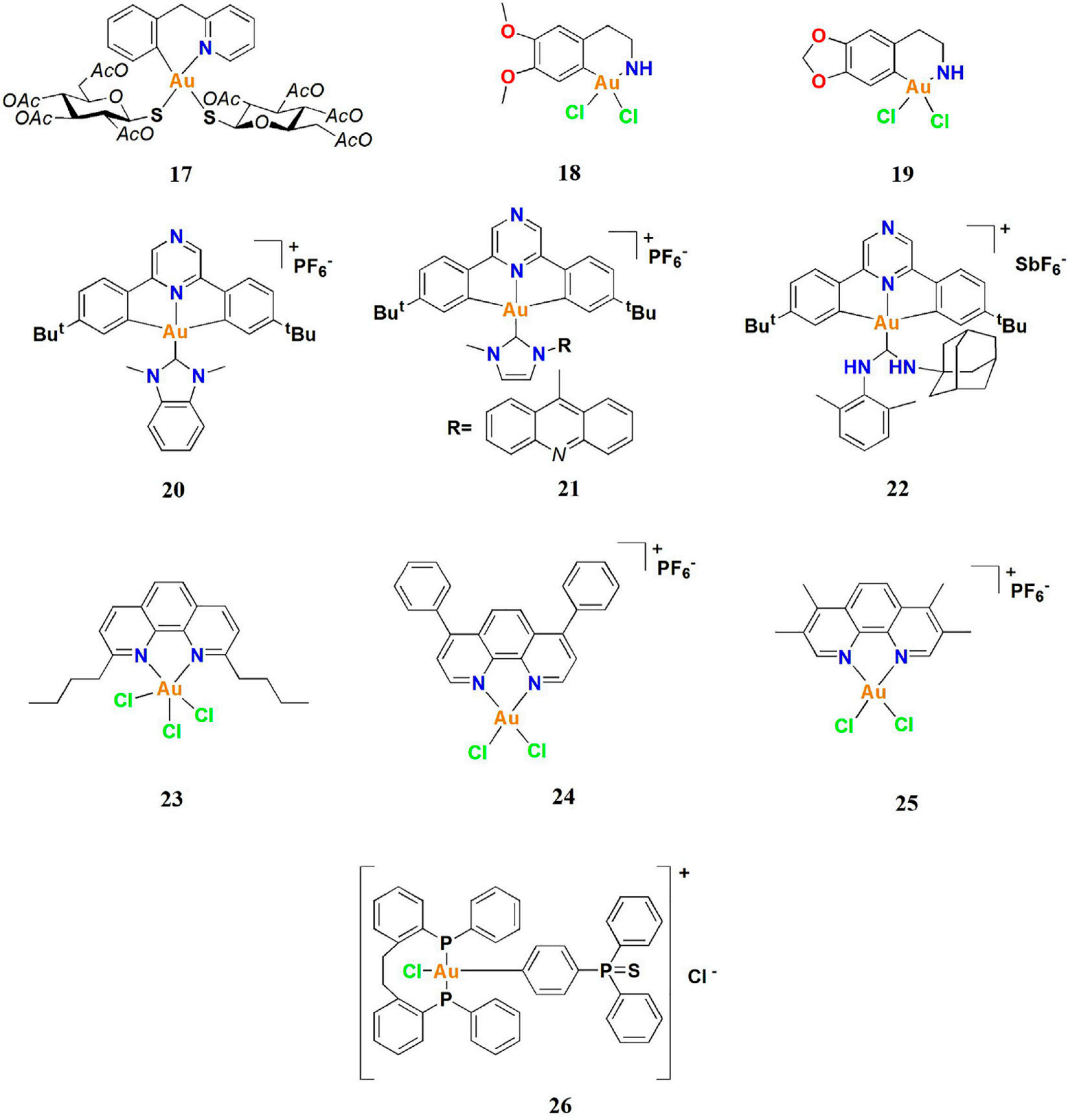
Anti-lung cancer gold (III) complexes.

Like many gold(I) complexes, gold (III) complexes can inhibit the activity of mercaptan-containing enzymes (including TrxR) by forming Au-S bonds through ligand exchange reactions (Messori et al., 2009). Nevertheless, there are also many excellent examples of physiologically stable gold (III) complexes, which are well-known to show higher anticancer activity *in vitro* and *in vivo* and induce cancer cell death possibly by multi-target mechanisms (Che et al., 2017; Fernandes et al., 2018; Isab et al., 2019; Bhargava et al., 2021; Maia et al., 2021; Fichna et al., 2022). Choosing the appropriate ligand to stabilize Au^3+^ ions is very important for cancer treatment (Che et al., 2010; Lin et al., 2010). Most of the reported anticancer gold (III) complexes focus on polydentate ligands, such as C ^ N, N ^C ^ N, N ^ N, C ^ N ^C, and phosphine (Figure 10). The Au^3+^ ions are highly stabilized under physiological conditions and have been reported to display potent anti-lung cancer activity.

### Anti-lung cancer gold (III)-(C^N) complexes

**FIGURE 11:**
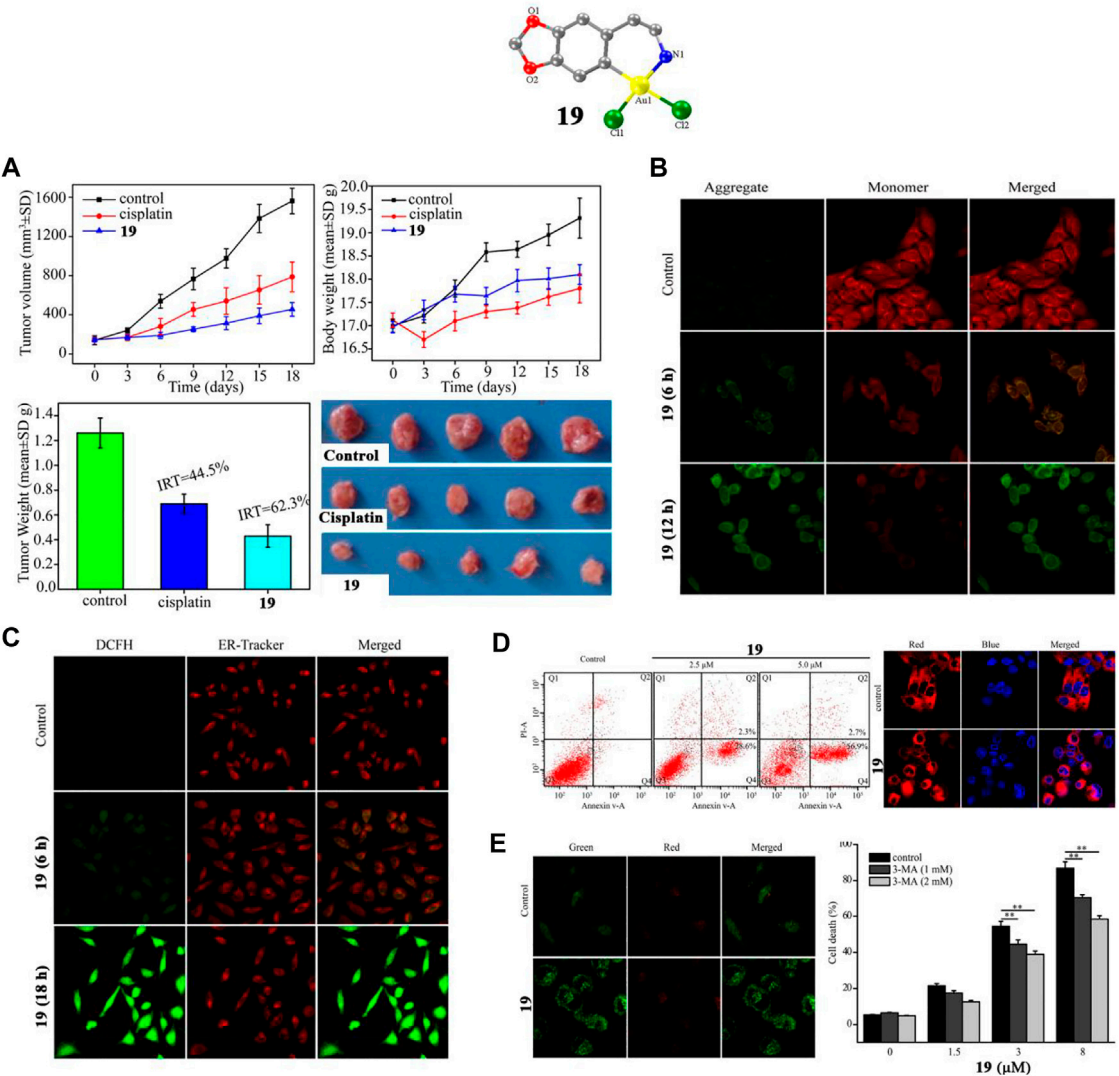
**(A)** BALB/c nude mice bearing A549 tumors after treatment with complex 1**9** (10 mg/kg/3 days) for 18 days. **(B)** Mitochondrial membrane potential in A549 cells after treatment with complex **19** at IC_50_ value for 0.5 h. **(C)** ROS production by ER-Tracker Red and H_2_DCFDA in A549 cells after treatment with complex **19** at IC_50_ value for 6 h. **(D)** A549 cell apoptosis after treatment with complex **19** at IC_50_ value measured by flow cytometer and confocal microscopy. **(E)** Analysis of autophagy in A549 lung cancer cells after treatment with complex **19** at IC_50_ value. Reproduced with permission (Liang et al., 2018).

Casini and co-workers described a gold (III)-(C^N) complex **17** by modifying auranofin with C^N cyclometalated 2-benzylpyridine in 2015. However, complex **17** did not display a lower IC_50_ value than auranofin (Casini et al., 2015). In 2018, Liang and co-workers described two similar tetrahydroisoquinoline gold (III)-(C^N) complexes **18** and **19** (Liang et al., 2018)**,** which induce endoplasmic reticulum stress-mediated apoptosis and pro-death autophagy in A549 lung cancer cells. The results suggesting that organometallic complexes in which an Au replaces the first CH_2_ group of the N-heterocyclic scaffolds of 1,2,3,4-Tetrahydroisoquinoline possess enhanced cytotoxic activities. Among these two gold complexes, complex **19** not only showed strong anti-proliferative ability in lung cancer cell lines, but was also equally potent in cisplatin-resistant lung cancer cell lines (A549 CDDP cells), which is indicative of a different mechanism than cisplatin. Complex **19** also showed strong anti-lung cancer ability *in vivo*. After intraperitoneal injections with cisplatin (3 mg/kg) or complex **19** (10 mg/kg) for 18 days in A549 xenograft tumor-bearing mice, complex 1**9** significantly inhibited tumor growth, leading to ∼62.3% reduction of tumor weight (Figure 11A), which was greater than that of cisplatin (∼44.5%). Liang and co-workers confirmed in A549 lung cancer cells that complex **19** could induce mitochondrial damage and causes ATP depletion, mitochondrial membrane depolarization (Figure 11B), elevated ROS levels (Figure 11C), endoplasmic reticulum stress, and ultimately induce apoptosis (Figure 11D) and pro-death autophagy (Figure 11E).

### Anti-lung cancer gold (III) (C^N^C) complexes

Bochmann and co-workers synthesized and characterized three pyrazine-based cyclometalated (C ^ N ^ C) gold (III) complexes in 2017 (Bochmann et al., 2017b). Of the series, the neutral alkynyl and thiophenolate complexes were nontoxic, whereas the benzimidazolylidene complex **20** showed strong antiproliferative activity, with the cell viability reduced to 22%. In addition, basis of the antiproliferative results, it might be suggested that when PF6 was incorporated into gold complexes, the resulting gold complexes tended to show an enhanced anticancer effect with fewer side effects *in vitro*. Complex **20** containing the benzimidazole-based NHC ligand had remarkable cytotoxicity, the IC_50_ values are in the micromolar to the submicromolar range and lower than the other two complexes and cisplatin. With cytotoxicity in the A549 cell line four times more than cisplatin, complex **20** was encouragingly effective. However, complex **20** also had the disadvantage of high cytotoxicity against healthy fibroblast MRC-5 cells. For higher cytotoxicity, 1,3-dimethylbenzimidazol-2-ylidene and pyrazine-based cyclometalated (C^N^C) ligand to stabilize the Au^3+^ ion is very important. The stable structure of complex **20** contributes to interacting more tightly with human telomerase G-quadruple DNA structures. Inhibition of the MDM2 interaction with p53 by complex **20** was also reported, providing new clues for the possible intracellular targets of this complex. From 2017 to 2018, Bochmann and co-workers tried to modify complex **20** and increase its cytotoxicity in A549 cells, such as complexes **21** and **22** (Bochmann et al., 2017a, 2018). Complex **21** with acridine functionality bound to the NHC ligand, it was more than 3–4 times more toxic than cisplatin against the A549. The complexes with the most lipophilic side-chains turned out to have a reduced activity compared to the other amino ester decorated complexes, while only the adamantyl derivative **22** was also more toxic than cisplatin against lung cancer cells, and the adamantyl compound **22** was over ten times more potent than Au-NHC. Among the series of amino ester derivatives, the glycinebased complex appeared as the least toxic in the series.

### Anti-lung cancer gold (III) (N^N) complexes

Many anticancer metal complexes with excellent cytotoxicity have been reported based on phenanthroline bidentate ligands. In 2014, Eichler and co-workers introduced a series of gold (III) (N^N) complexes based on phenanthroline ligands (Eichler et al., 2014). Compounds **23** was centered on the premise that the phenanthroline ligand would impart greater redox stability to the gold (III) center, which in turn would guard this class of metal-based drugs against inactivation/reduction by glutathione. Complex **23** has shown promising anti-lung cancer (A549) activity with a remarkable IC_50_ value, significantly higher than cisplatin in clinical use. In addition, complex **23** has been reported to effectively inhibit TrxR, even more than the known TrxR inhibitor aurothiomalate; therefore, complex **23** possibly induces lung cancer cell death via a TrxR-mediated mechanism.

As described earlier, gold (III) complexes may induce cancer cell death by multi-target mechanisms. Casini and co-workers have modified several gold (III) (N^N) complexes based on phenanthroline ligands as potential AQP3 inhibitors in 2019, including complexes **24** and **25** (Casini et al., 2019). Aquaporins (AQPs), such as aquaporin-3 (AQP3), which play crucial roles in cell apoptosis, proliferation, and migration, have been proposed as new drug targets for cancer treatment. The two complexes displayed potent cytotoxicity in the A549 cell line, which expresses AQP3, with IC_50_ values of 0.43 and 0.83 μM for **24** and **25**, respectively. Biophysical techniques and computational methods were used to investigate the mechanism of inhibition of AQP3 by gold (III) complexes. Complexes **24** and **25**, with EC_50_ values in the low micromolar range to AQP3, outperformed the benchmark compounds Auphen and Aubipy. This report indicated that in the future, gold (III) complexes based on phenanthroline ligands could be used as potential anticancer agents via AQP inhibition.

However, even though gold (III) complexes based on phenanthroline ligands have displayed potent anti-lung cancer activity *in vitro,* the *in vivo* anti-lung cancer efficacy remains in question. Eichler and co-workers demonstrated that those gold (III) complexes could interact with serum albumin proteins, which may cause the gold (III) complex to be destroyed during blood circulation.

### Anti-lung cancer gold (III)-phosphine complexes

**FIGURE 12:**
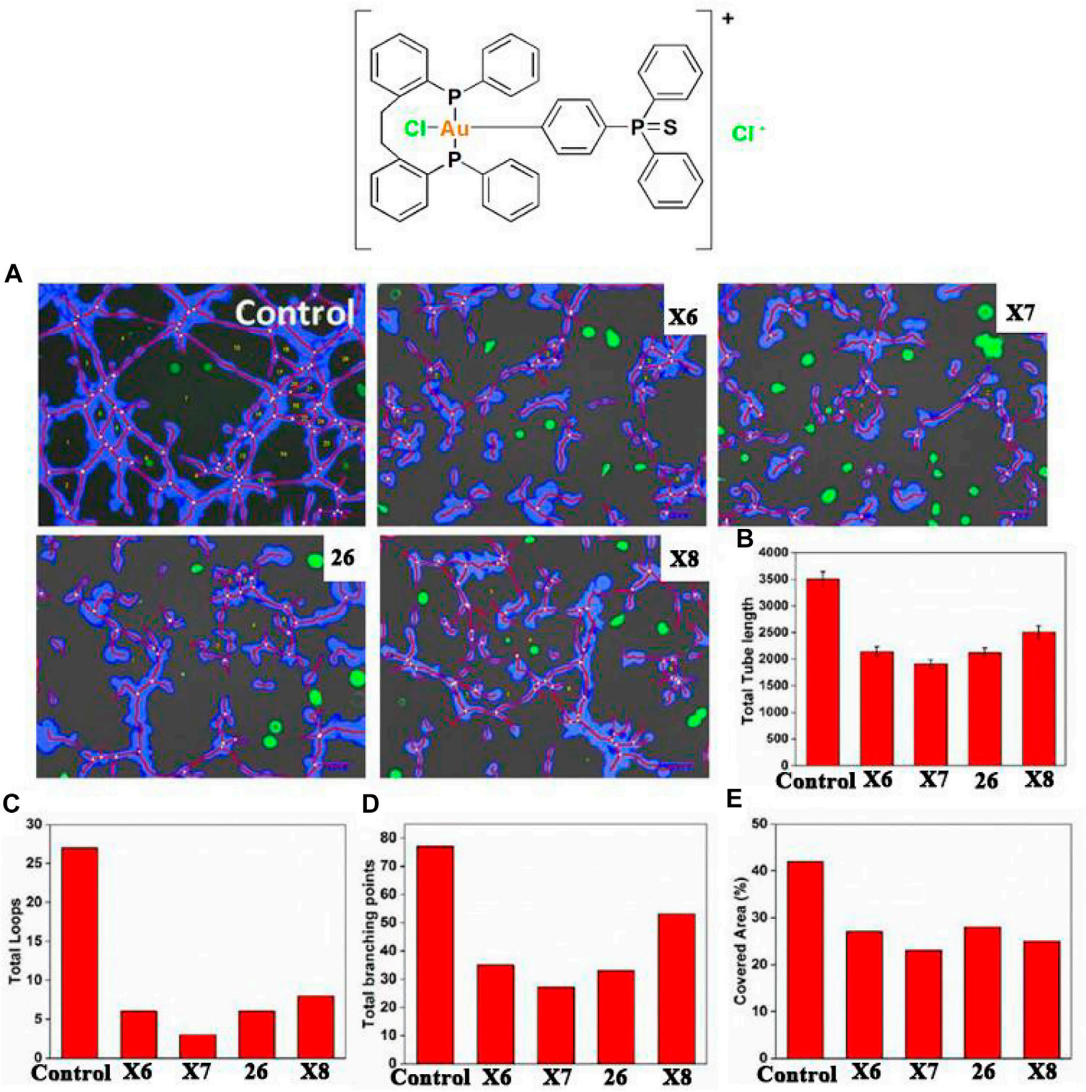
Tube formations of human umbilical vascular endothelial (HUVEC) cells. HUVECs cultured on Matrigel-coated plates and treated with gold (III)-phosphine complexes at IC_50_ concentrations for 16 h. **(A)** Tube formation observed by fluorescence microscopy. **(B–E)** The total lengths of the branching points, covered area, tubes, and loops were quantified. Reproduced with permission (Bhargava et al., 2018).

Gold (III)-phosphine complexes have also shown great potential in anti-lung cancer studies. Bhargava and co-workers developed a stable anti-lung cancer gold (III) complex **26** containing cyclometalated triphenylphosphine sulfide ligands in 2020 (Bhargava et al., 2018). Preliminary *in vitro* cytotoxicity studies found that complex **26** can inhibit A549 cells. Similarly, in reports by Mostafa and co-workers in 2019, complex **26** had significant antiangiogenic effects and also induced apoptosis by increasing ROS levels. Bhargava and co-workers investigated the angiogenesis-inhibiting properties of complex **26** using WimTube (Figure 12). Quantification revealed all aspects of network formation were significantly affected in complex **26** groups, indicating excellent antiangiogenic properties**.** Those results suggest cycloaurated gold (III) complexes may become new drug molecules for future cancer therapeutics.

## Approaches adopted to improve the efficiency of gold complexes against lung cancer

Although significant achievements have been made in developing anticancer gold complexes showing excellent stability and cytotoxicity *in vitro* and *in vivo*, poor bioavailability, selectivity, and serious toxicity have hindered their clinical application. Fortunately, various biocompatible nanoparticle (NP)-based drug-delivery systems, such as hydrogel (Zhang et al., 2019), endogenous protein (Zhang et al., 2020; Zhang et al., 2021b), liposomes (Liu et al., 2020), and silica (Che et al., 2014; Zhang et al., 2021b) have been reported for metal complexes targeting cancer treatment. Furthermore, the use of drug carriers has recently been described to enhance the anti-lung cancer effects of gold complexes.

**FIGURE 13:**
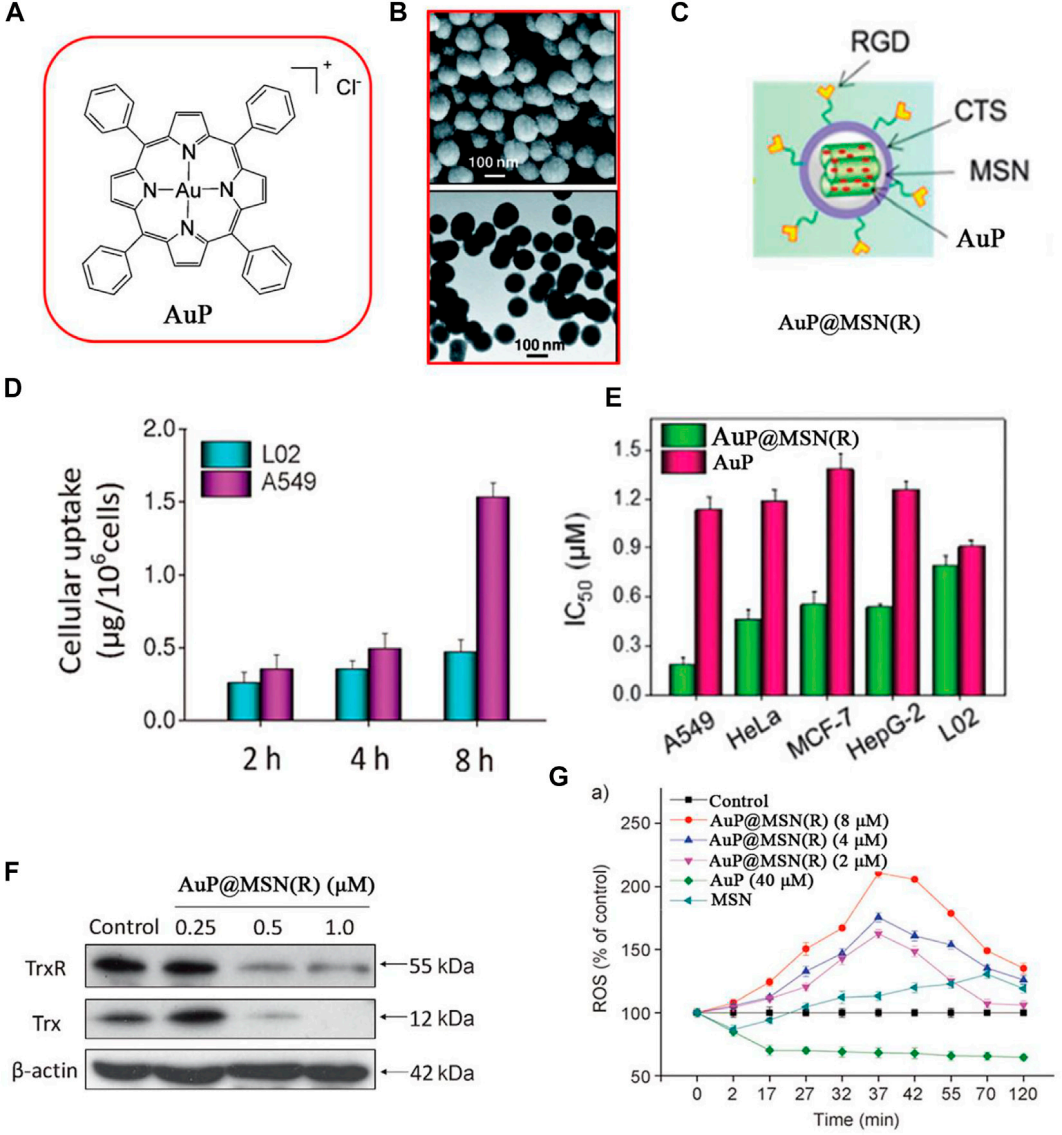
**(A)** Chemical structure of AuP. **(B)** SEM and TEM of AuP@MSN(R). **(C)** Structure of AuP@MSN(R). **(D)** Cytotoxicity of AuP@MSN(R) and AuP against cancer cells. **(E)** Cellular uptake of AuP@MSN(R) in A549 lung cancer cells and L02 normal cells. **(F)** Western blot of TrxR in A549 cells after being treated with AuP@MSN(R) for 24 h. **(G)** Increase intracellular ROS levels treated with AuP@MSN(R), AuP, and MSN. Reproduced with permission (Che et al., 2014).

In 2014, Che and co-workers used low toxicity mesoporous silica nanoparticles (MSNs) to enhance the anticancer efficacy of gold (III) porphyrin complex (AuP) (Figures 13A–C) (Che et al., 2014). The RGD peptide modified on the surface of MSNs effectively enhanced the cellular uptake of AuP and decreased the damage to normal cells. When the cellular uptake of AuP was analyzed by inductively coupled plasma mass spectrometry (ICP-MS), the results showed that intracellular Au content increased in a time-dependent manner in A549 cells but had a lower accumulation in L02 cells (Figure 13D). Encapsulation of AuP to form AuP@MSN(R) significantly improved the cytotoxicity of AuP to A549 cells (Figure 13E) and inhibitory effects on TrxR (Figure 13F). Compared to free AuP, AuP@MSN(R) could induce higher ROS levels and oxidative stress in cancer cells (Figure 13G) and activate ROS-mediated apoptosis signaling pathways.

**FIGURE 14:**
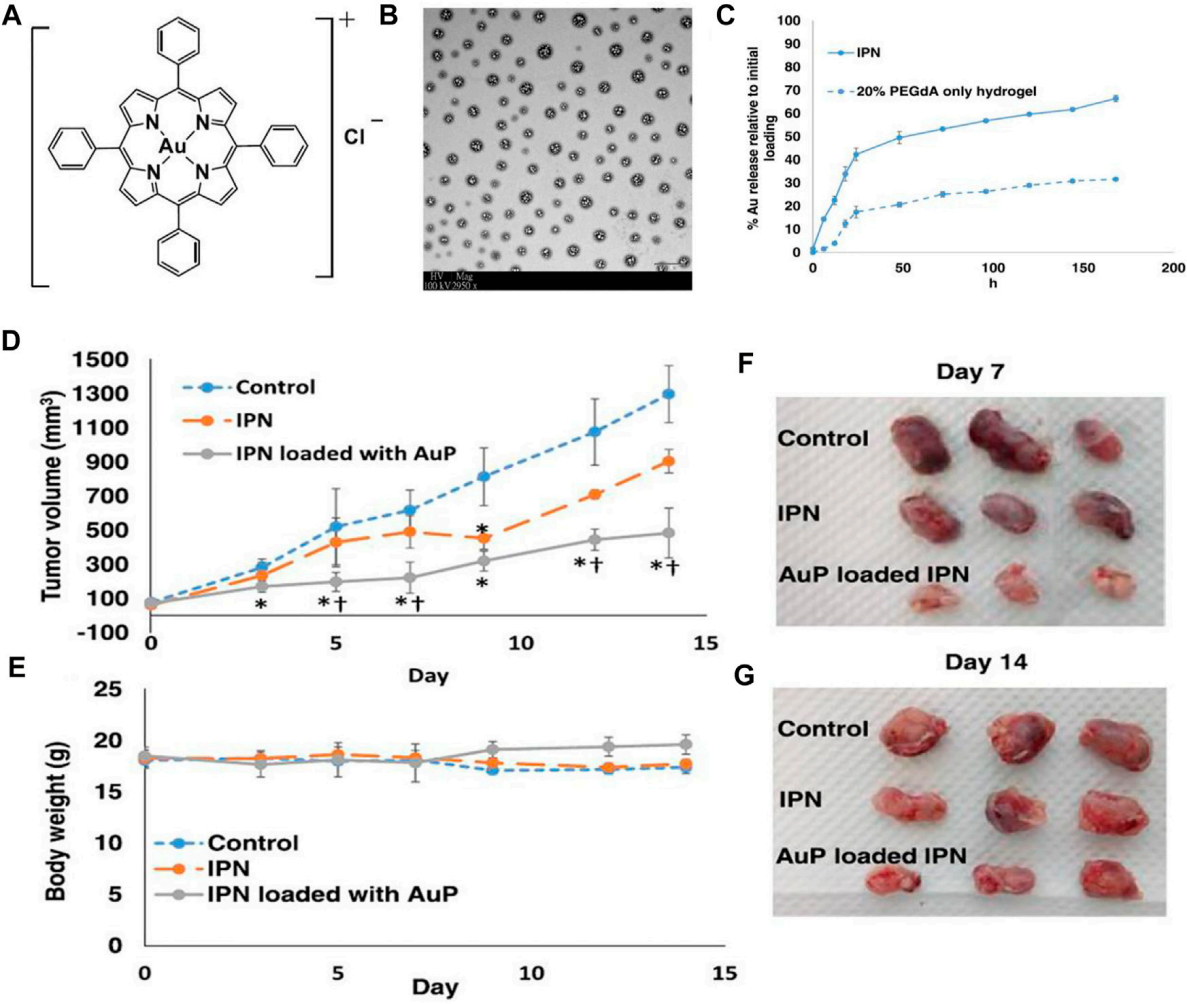
**(A)** Gold (III) porphyrin (AuP). **(B)** TEM image of hydrogel, scale bar 1 μm. **(C)** The release kinetics of AuP from hydrogel to 7 days *in vitro*. **(D–G)** Tumor growth inhibition in lung cancer xenografted mice for 14 days of treatment. Reproduced with permission (Che et al., 2019, 2020).

Another work by Che and co-workers reported the bioavailable and biodegradable hydrogel formulation using an interpenetrating network (IPN) matrix to deliver AuP for lung cancer treatment (Figures 14A–G) (Che et al., 2019, 2020). Xenograft model tests indicated that hydrogel improved the AuP *in vivo* anticancer efficacy compared to free AuP (Figures 14D–G) and effectively reduced systemic toxicity. The biocompatible hydrogel that can selectively accumulate in tumor tissue and can locally release AuP has promising chemotherapeutic potential. Similar delivery systems used to deliver other gold complexes for the treatment of lung cancer display excellent preclinical applications.

## Conclusion and prospects

In recent years, gold complexes have been developed as potential anti-lung cancer drugs in the hope of solving the resistance problem of cisplatin. Thiolenzymes, such as thioredoxin reductase (TrxR), are generally considered key targets of anti-lung cancer gold(I) and gold (III) complexes due to the high binding affinity of Au ions with thiols. The X-ray crystal structures and “omics” technologies provide valuable information for gold–protein interactions in the literature, significantly contributing to determining anticancer targets and pathways of gold complexes.

The cytotoxicity and molecular mechanisms of gold(I) complexes against lung cancer cells, which mainly inhibit the activity of thiol enzymes, seem clear. Obviously, gold (III) complexes can also inhibit the activity of thiol enzymes (including TrxR) by forming Au-S bonds similar to most gold(I) complexes. Nevertheless, evidence confirms that physiologically stable gold (III) complexes show higher anti-lung cancer activity *in vitro* and *in vivo* by multi-target mechanisms. Such mechanisms include the induction of mitochondrial damage causing ATP depletion, mitochondrial membrane depolarization, increased intracellular ROS levels, endoplasmic reticulum stress, and ultimately apoptosis and autophagy in lung cancer cells. Or they may form adducts with other anticancer molecular targets and interfere with cellular signaling pathways.

In addition, novel drug carriers will improve *in vivo* delivery efficiency, bioavailability, and targeting, while reducing unexpected side effects. In summary, with appropriately designed ligands to gold (III)/gold(I) complexes and utilization of drug micro-carriers to improve stability and efficacy, it is feasible to develop anti-lung cancer gold complexes with promising chemotherapeutic potential.
